# Multi-Type Sensor Placements in Gaussian Spatial Fields for Environmental Monitoring †

**DOI:** 10.3390/s19010189

**Published:** 2019-01-07

**Authors:** Chenxi Sun, Yangwen Yu, Victor O. K. Li, Jacqueline C. K. Lam

**Affiliations:** Department of Electrical and Electronic Engineering, The University of Hong Kong, Hong Kong, China; cxsun@eee.hku.hk (C.S.); ywyu@eee.hku.hk (Y.Y.); jcklam@eee.hku.hk (J.C.K.L.)

**Keywords:** multi-type sensor placement, submodular optimization, gaussian process

## Abstract

As citizens are increasingly concerned about the surrounding environment, it is important for modern cities to provide sufficient and accurate environmental information to the public for decision making in the era of smart cities. Due to the limited budget, we often need to optimize the sensor placement in order to maximize the overall information gain according to certain criteria. Existing work is primarily concerned with single-type sensor placement; however, the environment usually requires accurate measurements of multiple types of environmental characteristics. In this paper, we focus on the optimal multi-type sensor placement in Gaussian spatial field for environmental monitoring. We study two representative cases: the one-with-all case when each station is equipped with all types of sensors and the general case when each station is equipped with at least one type of sensor. We propose two greedy algorithms accordingly, each with a provable approximation guarantee. We evaluated the proposed approach via an application in air quality monitoring scenario in Hong Kong and experimental results demonstrate the effectiveness of the proposed approach.

## 1. Introduction

Environmental monitoring plays an essential role in the era of smart cities [1,2], providing not only sufficient information for citizens’ decision making, such as whether the air quality is suitable for exercise outdoors, but also as the primary data source for many longitudinal environment and health studies in order to better understand and assess the environment dynamics and their impact on public health over time [3,4,5]. However, deploying fixed-location sensors or monitoring stations that could provide accurate and calibrated measurements are costly, including not only the sensor cost which can be up to € 30,000 per device [6], but also the operation cost and site construction cost [7]. Recent studies have shown that low-cost sensors are not yet ready for providing accurate measurements [6] despite their recent popularity. Therefore, there is usually a fixed budget for deploying the sensors and locations should be chosen carefully according to certain objectives, leading to the sensor placement problem.

The general sensor placement problem has been studied in many environmental monitoring applications, for example, temperature monitoring [8], water contamination [9], wind monitoring [10], soil moisture [11], etc. In these previous work, the underlying spatial field, sometimes after preprocessing step, such as time series segmentation [10] or log-transformation [8], are modeled by the Gaussian Process (GP), which is a powerful probabilistic framework for modeling spatial phenomena and allows information theoretic criteria such as minimum conditional entropy and maximum mutual information to be applied for finding the most informative locations.

**Figure 1:**
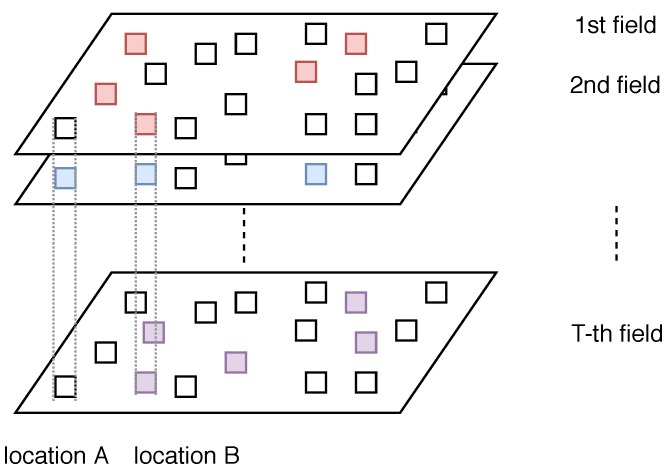
Illustration of the challenge of multi-type sensor placement.

Unfortunately, most of the existing work focus on single-type sensor placement problem under cardinality/budget constraints while some complex environment phenomena require measurements of multiple types of spatial fields simultaneously. A motivating scenario is to monitor the air quality of a region which requires measurements of multiple types of atmospheric pollutants. Take China as an example, six types of pollutants are measured [7], namely nitrogen dioxide (${\mathrm{NO}}_{2}$), carbon monoxide (CO), sulfur dioxide (${\mathrm{SO}}_{2}$), finite suspended matter (${\mathrm{PM}}_{2.5}$), respirable suspended particulates (${\mathrm{PM}}_{10}$) and ground level ozone ($O_{3}$). The air quality metric varies with different countries. In China, these six pollutants are measured to calculate the Air quality index (AQI) level. In the United States, ${\mathrm{PM}}_{2.5}$ and ${\mathrm{PM}}_{10}$ are considered together as particulate matter. Since each field is likely to exhibit different spatial patterns, it is not cost effective to apply the single-type sensor placement strategies for each field which may lead to a waste of site construction cost (around 200k USD) and maintenance cost (around 30k USD per year) [12] for deploying only one type of sensor. Figure 1 further illustrates the challenge of multi-type sensor placement problem. Suppose that there are *T* types of fields of interest in total. For each field, the five colored grids denote the optimal single-type sensor placement result (the five most informative locations). In particular, Location B is selected for all types for fields, while Location A is selected only for the second field. Therefore, when considering all the fields at the same time, selecting Location A for deploying a station may not be a good choice when the budget is limited and a careful design of placement scheme is required to balance the trade-off between information gain and cost.

There are some studies that investigated the multi-type sensor placement problem. Singh et al. [13] studied optimal sensor placement of two types of sensors that differ in cost and coverage with a total budget constraint. Ohsaka and Yoshida [14] gave a formulation for the joint placement of *k* types of sensors under the individual set size constraints and the total size constraints. However, both studies assume that each location can only install one type of sensor, yet in reality different types of sensors can be integrated together as a modular sensor box [15] and monitoring stations are often equipped with more than one type of sensors [16]. Yuen and Kuok [17] proposed a Bayesian sequential multi-type sensor placement algorithm for structural health monitoring and Lin et al. [18] proposed a non-dominated sorting genetic algorithm for solving the optimal multi-type sensor placement for structural damage detection. However, both approaches are heuristic optimization methods with no approximation guarantee. Furthermore, they both require explicit bounds on the number of sensors for each type.

In terms of sensor placement problem for air quality monitoring, Hsieh et al. [12] proposed an entropy-minimization model to recommend the best locations for establishing new monitoring stations. However, the placement result depends on the accuracy of their proposed inference model and the model is only able to predict the AQI value of one type of pollutant (either ${\mathrm{PM}}_{2.5}$ or ${\mathrm{PM}}_{10}$) rather than the general AQI, which better reflects the air quality.

In this paper, we study the optimal budgeted multi-type sensor placement problem without the disjoint assumption. Our major contributions are as follows. Firstly, we formulate the optimal multi-type sensor placement problem for environmental monitoring under a general budget constraint. Next, we investigate two representative scenarios: the one-with-all case that installs all types of sensors for each placement and the general case that only requires at least one type of sensor to be installed at each location. By exploiting the nice monotonicity and submodularity, we propose two greedy algorithms for solving the corresponding scenarios with provable approximation guarantee. We adapt the lazy approach for the two proposed greedy algorithms, which can further speed up the performance without hurting the approximation guarantee. Finally, we perform a case study using the air quality measurements in year 2017 from the official government stations of Hong Kong to demonstrate the proposed approach. This formulation provides guidance for city planners to design the multi-type sensor network for environmental monitoring.

The rest of the paper is organized as follows. In Section 2, we review the Gaussian Process (GP) model and optimal design criteria for single spatial field, and introduce the optimal multi-type sensor placement problem. In Section 3, we study the problem in the one-with-all case and the general case, and provide greedy algorithms with approximation guarantees. In Section 4, we provide the simulation results on the Hong Kong air quality monitoring data. We conclude in Section 5.

## 2. Problem Formulation

In this section, we review the relevant background including Gaussian Process and informative location for single spatial field, and formally formulate the optimal multi-type sensor placement problem. The major notations are summarized in Table 1.

### 2.1. Gaussian Process

To quantify the information gain by the placement, we adopt the Gaussian Process (GP) representation for the spatial fields. Gaussian Process is a powerful framework for making probabilistic predictions of spatial phenomena [19]. Intuitively, it generalizes multivariate Gaussian to an infinite number of random variables such that the joint distribution over every finite subset of random variables follows a Gaussian distribution.

Each GP GP(m(·),k(·,·)) is fully specified by a mean function m(·) and a symmetric positive-definite covariance function (also known as kernel) k(·,·). An important property of GP is that, for every finite subset *A* of the index set *V*, the joint distribution over these random variables $X_{A}$ is Gaussian. Then, for the random variable with index *u*, its mean $\mu_{u}$ is given by m(u). For each pair of random variables with indexes u,v, their covariance $\sigma_{u,v}$ is given by k(u,v). We denote the mean vector of the set of random variables *A* by $\mu_{A}$ and their covariance matrix by $\Sigma_{AA}$.

Let V:={1,2,⋯,n} denote a finite set of indexes, each corresponding to a location (square grid) of the city region. Let $\mathrm{GP}(m_{i}\left(\cdot \right),k_{i}\left(\cdot ,\cdot \right))$ denote the Gaussian Process for the *i*th spatial field and *T* denote the types of spatial fields of interest. Let [T]:={1,2,⋯,T}. Suppose for the *i*th spatial field, e.g. ${\mathrm{NO}}_{2}$, a set of observations $X_{A}=x_{A}$ corresponding to the finite subset A⊂V can be obtained either through pre-deployments or mathematical simulations, we can then predict the value of any point y∈V. By definition, the distribution of $X_{y}$ given these observations is a Gaussian whose conditional mean $\mu_{y|A}$ and variance $\sigma_{y|A}^{2}$ are given by:

(1) $$\begin{array}{c} \mu_{y|A}=m_{i}\left(y\right)+\Sigma_{yA}\Sigma_{AA}^{-1}\left(x_{A}-\mu_{A}\right), \end{array}$$

(2) $$\begin{array}{c} \sigma_{y|A}^{2}=k_{i}\left(y,y\right)-\Sigma_{yA}\Sigma_{AA}^{-1}\Sigma_{Ay}, \end{array}$$

where $\Sigma_{yA}$ is a covariance vector with one entry for each u∈A with value $k_{i}\left(y,u\right)$ and $\Sigma_{Ay}=\Sigma_{yA}^{T}$.

To compute the predictive distribution above, we need to know the mean and covariance functions. The mean function can be estimated by regression. The covariance function, depending on the specific scenarios, can be obtained by either learning the hyperparameters of some existing family of kernel functions [19] such as Gaussian kernel $k_{\theta }\left(d\right)=\exp\left(-\frac{d^{2}}{\theta^{2}}\right)$ with hyperparameter θ, or learning complex nonstationary kernels from sensory data collected by pre-deployment [20].

### 2.2. Informative Locations for Single Spatial Field

There are two common criteria for deciding what a good design is for placing single-type sensors in Gaussian Process: entropy [21] and mutual information [8]. Entropy criterion seeks to place sensors at the most uncertain places so as to minimize the conditional entropy of the unobserved locations V\A after placing sensors at locations *A*. Specifically, if the budget allows for *k* sensors in total, then we aim to find

(3) $$\begin{array}{c} A^{*}=\arg\min_{A\subset V:|A|=k}H\left(X_{V\backslash A}|X_{A}\right) \end{array}$$

$H(X_{V\backslash A}|X_{A})$ is the conditional differential entropy given by:

(4) $$\begin{array}{c} H\left(X_{V\backslash A}|X_{A}\right)=-\int p\left(x_{V\backslash A},x_{A}\right)\log p\left(x_{V\backslash A}|x_{A}\right)dx_{V\backslash A}dx_{A} \end{array}$$

where $p(x_{V\backslash A},x_{A})$ is the joint probability density function. Since $H\left(X_{V\backslash A}|X_{A}\right)=H\left(X_{V}\right)-H\left(X_{A}\right)$ where $H(X_{V})$ is invariant with respect to the choice of *A*, the optimization in Equation (3) is equivalent to

(5) $$\begin{array}{c} A^{*}=\arg\max_{A\subset V:|A|=k}H\left(X_{A}\right) \end{array}$$

The mutual information criterion on the other hand seeks to place sensors at locations *A* that most significantly reduce the uncertainty about the estimates in the rest of the space V\A. Specifically, if the budget allows for *k* sensors in total, then we aim to find

(6) $$\begin{array}{c} A^{*}=\arg\max_{A\subset V:|A|=k}I\left(X_{V\backslash A};X_{A}\right) \end{array}$$

where $I(X_{V\backslash A};X_{A})$ is the mutual information between the unknown locations $X_{V\backslash A}$ and the known locations $X_{A}$ which is given by

(7) $$\begin{array}{c} I\left(X_{V\backslash A};X_{A}\right)=H\left(X_{V\backslash A}\right)-H\left(X_{V\backslash A}|X_{A}\right) \end{array}$$

Finding the optimal solution of both optimizations in Equations (5) and (6) has been shown to be NP-hard [8,22]. Fortunately, both objective functions have the nice *monotone* and *submodular* properties (the mutual information is usually monotone and submodular under the assumption that k≪n).

**Definition** **1** (Non-decreasing)**.** *A set function $f:2^{V}\to R$ is called non-decreasing if for all A⊆B⊆V, we have*

$$\begin{array}{c} f(B)\geq f(A) \end{array}$$

**Definition** **2** (Submodularity)**.** *A set function $f:2^{V}\to R$ is called submodular if for all A⊆B⊆V and s∈V\B, we have*

$$\begin{array}{c} f(A\cup \{s\})-f(A)\geq f(B\cup \{s\})-f(B) \end{array}$$

Submodularity is also known as the diminishing returns property. Intuitively, the more sensors placed, the less information gain we can have by deploying a new sensor. An equivalent definition is as follows. A set function $f:2^{V}\to R$ is called submodular if for all A,B⊆V, we have

$$\begin{array}{c} f(A)+f(B)\geq f(A\cup B)+f(A\cap B) \end{array}$$

The choice of the informative criterion depends on the actual scenario. For now, we denote the general information gain of choosing the set of indexes A⊆V for deploying a certain type of sensors (using either criterion) as f(A). Then, both optimizations in Equations (5) and (6) can be written as the following submodular function maximization:

(8) $$\begin{array}{c} A^{*}=\arg\max_{A\subset V:|A|=K}f\left(A\right) \end{array}$$

where *K* is subset size constraint (also known as the cardinality constraint), i.e., the total number of sensors we can place due to the total budget constraints.

Despite the hardness of the optimization above, the nice monotone and submodular properties allow us to solve the problem via the simple greedy algorithm with provable approximation guarantee. The algorithm starts with the empty set and then at each iteration adds to the current set *A* the index *s* that maximizes the incremental information gain f(A∪{s})−f(A) and continues until the subset size constraint |A|≤K is no longer satisfied.

**Theorem** **1** ([23])**.** *If the submodular set function f is monotone and f(∅)=0, then the greedy algorithm finds a solution A such that $f\left(A\right)\geq \left(1-1/e\right)f\left(A^{*}\right)$ with at most O(K|V|) evaluations of f*.

It is not hard to know that f(∅)=0. Hence, by this theorem, we know that the greedy algorithm gives an approximation ratio of 1−1/e≈0.632 for the single-type sensor placement problem.

### 2.3. Optimal Multi-Type Sensor Placement

In many cases, we would like to place multiple types of sensors simultaneously for monitoring a complex spatial phenomena given a fixed budget. A motivating scenario is to monitor the air quality of a region which requires measurements of six types of atmospheric pollutants [7], namely nitrogen dioxide (${\mathrm{NO}}_{2}$), carbon monoxide (CO), sulfur dioxide (${\mathrm{SO}}_{2}$), finite suspended matter (${\mathrm{PM}}_{2.5}$), respirable suspended particulates (${\mathrm{PM}}_{10}$) and ground level ozone ($O_{3}$).

**Figure 2:**
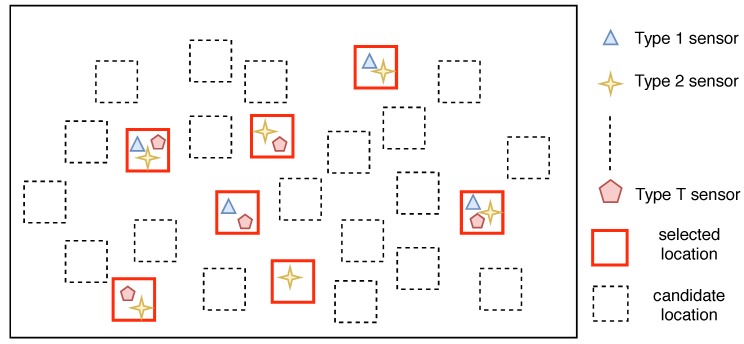
An example of the multi-type sensor placement scheme.

Figure 2 shows an example of the multi-type sensor placement scheme. The red rectangles denote the selected locations for deploying stations. For each station, one or multiple types of sensors are installed to monitor the spatial fields. Specifically, the optimal multi-type sensor placement problem aims to figure out where are the best locations for deploying the stations and what type of sensors should be installed at each location in order to achieve certain objectives.

Let $f_{i}\left(A_{i}\right)$ denote the information gain of choosing the set of indexes (locations) $A_{i}\subseteq V$ for deploying the *i*th type of sensors. For simplicity of notation, let $A:=\{A_{1},A_{2},\cdots ,A_{T}\}$ denote the multi-type sensor placement scheme.

Now, we proceed to discuss the cost function for the multi-type sensor placement case. An important observation is that, when multiple types of sensors are placed together, the total cost is smaller than the sum of individual cost due to the existence of site construction cost and site operation cost (http://aqicn.org/products/monitoring-stations/).

Let $c_{i}$ denote the equipment cost for the *i*th type which includes both the initial cost and the sensor-specific operation cost (e.g., calibration cost, sampling cost, etc.), then the total equipment cost $c_{e}\left(A\right)$ can be expressed as

(9) $$c_{e}\left(A\right)=c_{1}\cdot |A_{1}|+c_{2}\cdot |A_{2}|+\cdots +c_{T}\cdot \left|A_{T}\right|$$

Let $c_{s}\left(A\right)$ denote the site cost which includes the construction cost and site operation cost. If we assume that the site cost is invariant with the location, i.e. $c_{site}$ for each station, $c_{s}\left(A\right)$ can be expressed as

(10) $$c_{s}\left(A\right)=c_{site}\cdot \left|A_{1}\cup A_{2}\cup \cdots \cup A_{T}\right|$$

Then, the cost function for A can be expressed as

(11) $$c\left(A\right)=c_{s}\left(A\right)+c_{e}\left(A\right)$$

and we aim to find

(12) $$\begin{array}{cc} A^{*}= & \arg\max_{c(A)\leq B}\;\left(f_{1}\left(A_{1}\right),f_{2}\left(A_{2}\right),\cdots ,f_{T}\left(A_{T}\right)\right) \end{array}$$

where *B* is the total budget constraint.

## 3. Solution Approach

In this section, we investigate two reasonable scenarios of the optimal multi-type sensor placement problem and propose two greedy approaches with provable approximation guarantees.

### 3.1. One-with-All Case

We start with the simplest condition where each station is equipped with all types of sensors. Let $c_{all}:=c_{site}+\sum_{i=1}^{T}c_{T}$ denote the cost of a station (with all type of sensors). In this case, we have $A_{1}=A_{2}=\cdots =A_{T}$ and the general cost constraint reduces to the cardinality constraint $\left|A\right|\leq \lfloor \frac{B}{c_{all}}\rfloor$ where ⌊x⌋ denotes the floor function mapping *x* to the greatest integer less than or equal to *x*. Let $A:=A_{1}=A_{2}=\cdots =A_{T}$ denote the placement scheme and *K* denote the total number of stations we can deploy. Then, the problem becomes:

(13) $$\begin{array}{cc} A^{*}= & \arg\max_{|A|\leq K}\;\left(f_{1}\left(A\right),f_{2}\left(A\right),\cdots ,f_{T}\left(A\right)\right) \end{array}$$

However, the optimal solution of the above multi-objective optimization does not exist. The reason is that different pollutant fields are likely to exhibit different spatial patterns due to different generation process.

**Figure 3:**
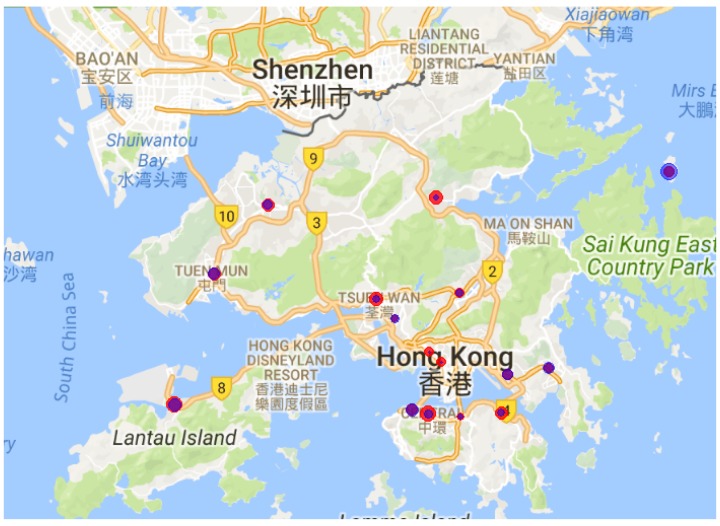
Spatial variations of the air quality measurements at the 16 official monitoring stations in Hong Kong in 2017: the blue circles denote ${\mathrm{NO}}_{2}$ and the red circles denote ${\mathrm{PM}}_{2.5}$. The size of a circle represents the magnitude of the variance of the corresponding random variable.

Figure 3 visualizes the different spatial characteristic of ${\mathrm{NO}}_{2}$ and ${\mathrm{PM}}_{2.5}$ in Hong Kong. The variance of the random variable at each monitored location is estimated with the hourly measurement data during Year 2017. As shown in Figure 3, the high variance locations with respect to ${\mathrm{PM}}_{2.5}$ are not always the locations where the variances are high with respect to ${\mathrm{NO}}_{2}$.

Instead, we can always find the Pareto-optimal solutions [24] of the above multi-objective problem. We say a placement scheme *A* is Pareto optimal if there is no other scheme $A^{\prime }$ such that $f_{i}\left(A^{\prime }\right)\geq f_{i}\left(A\right)$ for all *i* and $f_{j}\left(A^{\prime }\right)>f_{j}\left(A\right)$ for some *j*. In other words, *A* is Pareto-optimal if there is no other placement scheme that is no worse than *A* in all objectives and is strictly better than *A* in at least one objective $f_{j}$.

One standard approach to find such solutions is the weighted sum transformation/scalarization [24]:

(14) $$\begin{array}{cc} A^{*}= & \arg\max_{|A|\leq K}\;\sum_{i=1}^{T}w_{i}f_{i}\left(A\right) \end{array}$$

where $w_{i}>0$ denotes the weight parameter of the *i*th objective function and we have $\sum_{i=1}^{T}w_{i}=1$. By default, we can choose $w_{1}=w_{2}=\cdots =w_{T}=\frac{1}{T}$. Since submodularity is closed under linear combinations, the new objective function is also submodular. Hence, we can use the greedy approach to solve the problem for this case. The detail is summarized in Algorithm 1.

**Algorithm 1** Multi-type sensor deployment algorithm for one-with-all case
**Input:** Station number constraint *K*, a set of grids *V*, objective function $f_{1},f_{2},\cdots ,f_{T}$, weights $w_{1},w_{2},\cdots ,w_{T}$**Output:** A subset of locations A⊆V A=∅ **while** |A|≤K **do**  select location *s* that has the highest incremental gain $\sum_{i=1}^{T}w_{i}\left(f_{i}\left(A\cup \left\{s\right\}\right)-f_{i}\left(A\right)\right)$  add *s* to location set *A* **end while** **return** *A*

**Proposition** **1.** *Algorithm 1 finds a solution A such that $\sum_{i=1}^{T}w_{i}f_{i}\left(A\right)\geq \left(1-1/e\right)\sum_{i=1}^{T}w_{i}f_{i}\left(A^{*}\right)$ where $A^{*}$ is the Pareto-optimal solution with weight parameters $w_{1},w_{2},\cdots ,w_{T}$*.

Using the fact that the objective function is monotone and submodular, it directly follows from Theorem 3.

### 3.2. General Case

A more general scenario is when each station is only required to install at least one sensor. Take the weather monitoring stations in Hong Kong for example, many stations only contain some of the sensors, such as temperature, pressure, rainfall, etc. In this case, increasing the information gain of one type of sensor will decrease the information gain in another due to the total budget constraint. Hence, we adopt a similar weighted sum transformation approach and aim to solve the follow optimization:

(15) $$\begin{array}{cc} A^{*}= & \arg\max_{c(A)\leq B}\;\sum_{i=1}^{T}w_{i}f_{i}\left(A_{i}\right) \end{array}$$

where $w_{i}>0$ denotes the weight parameter of the *i*th objective function and we have $\sum_{i=1}^{c}w_{i}=1$.

To understand the difficulty of the optimization, we first investigate the structure of the cost constraint. Let $k_{i}\in N$ denote the number of sensors for the *i*th type and $K\in N^{+}$ denote the total number of stations of the placement scheme A. Then, the cost constraint can be rewritten as:

(16) $$\begin{array}{c} |A_{1}\cup A_{2}\cup \cdots \cup A_{T}|=K\in N^{+} \end{array}$$

(17) $$\begin{array}{c} |A_{i}|=k_{i}\in N,\;i\in \left[T\right] \end{array}$$

(18) $$\begin{array}{c} c_{site}\cdot K+\sum_{i=1}^{T}c_{i}\cdot k_{i}\leq B \end{array}$$

**Proposition** **2.** *Let $A^{*}$ denote the optimal placement scheme. If $K^{*}\geq T$ and $k_{i}^{*}\geq 1\;\forall i\in \left[T\right]$, when $\left\lfloor \frac{B-\sum_{i=1}^{T}\left(c_{i}-\min_{i}c_{i}\right)}{c_{site}+\min_{i}c_{i}}\rfloor =\lfloor \frac{B}{c_{all}}\right\rfloor$, the cost constraints in Equations (16)–(18) can be reduced to the cardinality constraint $k_{1}=k_{2}=\cdots =k_{T}=K=\lfloor \frac{B}{c_{all}}\rfloor$ for the optimal multi-type sensor placement*.

**Proof.**  Since the objective function $f_{i}$ is non-decreasing for each *i*, we aim to find the optimal integer solutions $\left(k_{1},k_{2},\cdots ,k_{T}\right)$ subject to the budget constraint in order the maximize the overall information gain.

Since the total sensor costs $\sum_{i=1}^{T}c_{i}\cdot k_{i}$ when there is *K* stations is at most $\sum_{i=1}^{T}c_{i}\cdot K$, we know that the achievable number of stations *K* is at least $k_{\min}:=\lfloor \frac{B}{c_{all}}\rfloor$ and equality is achieved when each station is equipped with all types of sensors (one-with-all case).

In the meantime, since $\sum_{i=1}^{T}c_{i}\cdot k_{i}$ is at least $\min_{i}c_{i}\cdot \left(K-T\right)+\sum_{i=1}^{T}c_{i}$ if we assume that there is at least one sensor for each type, we know that the achievable number of stations *K* is at most $k_{\max}:=\lfloor \frac{B-\sum_{i=1}^{T}\left(c_{i}-\min_{i}c_{i}\right)}{c_{site}+\min_{i}c_{i}}\rfloor$ and equality is achieved when each location is equipped with one sensor for each type, except for the cheapest type with (K−T+1) sensors.

Therefore, when $k_{\max}=k_{\min}$, *K* is unique and $k_{1}=k_{2}=\cdots =k_{T}=K$ can be achieved. Then, the cost constraints can be reduced to the cardinality constraint $k_{1}=k_{2}=\cdots =k_{T}=K=\lfloor \frac{B}{c_{all}}\rfloor$. ☐

**Remark** **1.** *The assumptions that K>T and $k_{i}\geq 1\;\forall i\in \left[T\right]$ is usually naturally satisfied with a reasonable budget that allows to place at least one sensor for each type of pollutant, as otherwise there be entirely no measurement for some types of field, making the total uncertainty still quite high*.

Proposition 2 gives the condition when the general case reduces to the one-with-all case. This usually happens when $c_{site}\gg c_{i}$ for all i∈[T]. In other words, when the sensor costs are negligible compared with the site construction costs, each station should be equipped with all types of sensors. The budget constraint limits the number of stations we can deploy.

In the following, we focus on the case when the cost constraint is not reducible to the one-with-all case. One might consider to greedily place sensors until the cost constraint can no longer be satisfied, i.e., at each step, we consider the location $s^{*}$ to place type $i^{*}$ sensor such that

(19) $$s^{*},i^{*}=\arg\max_{i,s}w_{i}\left(f_{i}\left(A_{i}\cup \left\{s\right\}\right)-f_{i}\left(A_{i}\right)\right)$$

and confirm the selection if its cost is acceptable. $A_{i}$ here refers to the current selected location set for the *i*th field. However, the solution can be arbitrarily bad as a sensor providing information gain *g* will always be preferred over a sensor providing information gain g−ϵ despite a much higher cost. Alternatively, we can consider to greedily assign sensors based on information gain per cost, i.e., at each step, we consider the location $s^{*}$ to place type $i^{*}$ sensor such that

(20) $$s^{*},i^{*}=\arg\max_{i,s}\frac{w_{i}\left(f_{i}\left(A_{i}\cup \left\{s\right\}\right)-f_{i}\left(A_{i}\right)\right)}{{△}_{i,s}c\left(A\right)}$$

and confirm the selection if its cost is acceptable. ${△}_{i,s}c\left(A\right)$ denotes its current cost. However, the solution can still be arbitrarily bad, as a cheap sensor ϵ with a higher information gain per cost (2ϵ/ϵ=2) will always be preferred over an expensive sensor *B* providing higher information gain *B* despite the remaining budget *B* only allows one to be selected and the better solution is to choose the expensive sensor.

Fortunately, the following theorem shows that the two solutions cannot be bad at the same time.

**Theorem** **2.** *Let $A_{G}$ denote the solution by greedy selection with the criteria in Equation (19) and $A_{CG}$ denote the solution by cost effective greedy selection with the criteria in Equation (20). If the submodular function $f_{1},f_{2},\cdots ,f_{T}$ are monotone and f(∅)=0, then we have*

$$\begin{array}{c} \max\left\{A_{G},A_{CG}\right\}\geq \frac{1}{2}\left(1-1/e\right)\max_{A:c(A)\leq B}\sum_{i=1}^{T}w_{i}f_{i}\left(A_{i}\right) \end{array}$$

**Proof.**  Let $\hat{V}:=\left\{1,2,\cdots ,nT\right\}$ denote a new set with $|\hat{V}\left|=T\cdot |V\right|$. Then, each placement scheme $A:=\{A_{1},A_{2},\cdots ,A_{T}\}$ corresponds to exactly one subset $\hat{A}\subseteq \hat{V}$ such that $\hat{A}=\left\{s+\left(i-1\right)\times T:\forall s\in A_{i}\;\forall i=\left[T\right]\right\}$. Let $f:2^{\hat{V}}\to R$ denote a set function such that $f\left(\hat{A}\right)=\sum_{i=1}^{T}w_{i}f_{i}\left(A_{i}\right)$. It is obvious that f(∅)=0.

We first show that *f* is non-decreasing. Let $\hat{B}\subseteq \hat{V}$ denote the corresponding set for placement scheme B. Since for all $\hat{A}\subseteq \hat{B}\subseteq \hat{V}$, we know that $A_{i}\subseteq B_{i}$ for all i∈[T], then $f\left(\hat{B}\right)=\sum_{i=1}^{T}w_{i}f_{i}\left(B_{i}\right)\geq \sum_{i=1}^{T}w_{i}f_{i}\left(A_{i}\right)=f\left(\hat{A}\right)$ and hence *f* is non-decreasing.

We then show that *f* is submodular. For $\hat{s}\in \hat{V}\backslash \hat{B}$, let j∈[T] denote its corresponding type and s∈V denote its corresponding location. Then, $f\left(\hat{B}\cup \left\{\hat{s}\right\}\right)-f\left(\hat{B}\right)=w_{j}\left(f_{j}\left(B_{j}\cup \left\{s\right\}\right)-f_{j}\left(B_{j}\right)\right)\geq w_{j}\left(f_{j}\left(A_{j}\cup \left\{s\right\}\right)-f_{j}\left(A_{j}\right)\right)=f\left(\hat{A}\cup \left\{\hat{s}\right\}\right)-f\left(\hat{A}\right)$ and hence *f* is submodular.

Therefore, *f* is non-decreasing and submodular with f(∅)=0 and by Theorem 3 in [9] which is a generalization of the Theorem in [25] for the special case of the budgeted max-cover problem, we know that $\max\left\{A_{G},A_{CG}\right\}\geq \frac{1}{2}\left(1-1/e\right)\max_{A:c(A)\leq B}\sum_{i=1}^{T}w_{i}f_{i}\left(A_{i}\right)$. The proof is now complete. ☐

Let V:={V,V,⋯,V} and ${△}_{i,s}c\left(A\right)$ denotes the incremental cost of adding type *i* sensor at location *s* when the existing placement scheme is A. Then, we know that

$${△}_{i,s}c\left(A\right)=\left\{\begin{array}{cc} ccc_{site}+c_{i} & \forall j\in \left[T\right]\;s\notin A_{j}\\ c_{i} & \mathrm{otherwise} \end{array}\right.$$

With the help of above additional notations, we now summarize the proposed hybrid greedy selection approach for the general multi-type sensor placement in Algorithm 2.

**Algorithm 2** Multi-type sensor deployment algorithm for the general cost case
**Input:** Budget *B*, a set of grid index *V*, objective function $f_{1},f_{2},\cdots ,f_{T}$, weights $w_{1},w_{2},\cdots ,w_{T}$, site construction cost $c_{site}$, sensor cost $c_{1},c_{2},\cdots ,c_{T}$**Output:** A placement scheme A⊂V $A^{\prime }=A^{\prime \prime }=\emptyset ,V^{\prime }=V^{\prime \prime }=V,B^{\prime }=B^{\prime \prime }=B$ **while** the search space $V^{\prime }$ is not empty **do**  Select location *s* for placing the *i*th type sensor that has the highest incremental gain $w_{i}\left(f_{i}\left(A_{i}^{\prime }\cup \left\{s\right\}\right)-f_{i}\left(A_{i}^{\prime }\right)\right)$  **if** the incremental cost ${△}_{i,s}c\left(A^{\prime }\right)$ is less than the remaining budget $B^{\prime }$ **then**   Update the remaining budget $B^{\prime }=B^{\prime }-{△}_{i,s}c\left(A^{\prime }\right)$ and add location *s* to the location set $A_{i}^{\prime }$  **end if**  Remove location *s* from search space $V_{i}^{\prime }$ **end while** **while** the search space $V^{\prime \prime }$ is not empty **do**  Select location *s* for placing the *i*th type of sensor that has the highest cost-effective gain $\frac{w_{i}\left(f_{i}\left(A_{i}^{\prime }\cup \left\{s\right\}\right)-f_{i}\left(A_{i}^{\prime }\right)\right)}{{△}_{i,s}c\left(A^{\prime \prime }\right)}$  **if** the cost ${△}_{i,s}c\left(A^{\prime \prime }\right)$ is less than the remaining budget $B^{\prime \prime }$ **then**   Update the remaining budget $B^{\prime \prime }=B^{\prime \prime }-{△}_{i,s}c\left(A^{\prime \prime }\right)$ and add location *s* to the location set $A_{i}^{\prime \prime }$  **end if**  Remove location *s* from search space $V_{i}^{\prime \prime }$ **end while** Select the better scheme $A=\arg\max_{A\in \{A^{\prime },A^{\prime \prime }\}}\sum_{i=1}^{T}w_{i}f_{i}\left(A_{i}\right)$ **return** A

**Proposition** **3.** *The time complexity of Algorithm 2 is $O(\left\lfloor \frac{B}{\min_{i}c_{i}}\right\rfloor T|V|)$*.

Sviridenko [26] showed that it is even possible to achieve the approximation ratio of 1−1/e for the general cost case; however, the algorithm requires an enumeration over all feasible sets of cardinality three and hence its complexity for our problem is $O(\left\lfloor \frac{B}{\min_{i}c_{i}}\right\rfloor T^{4}|V{|}^{4})$, which is impractical.

In many real cases, the bound is not tight. Hence, we also provide a tighter online bound for arbitrary placement scheme derived with the submodularity property.

**Theorem** **3** (Online bound)**.** *For a given placement scheme $\tilde{A}=\left\{\tilde{A_{1}},\tilde{A_{2}},\cdots ,\tilde{A_{c}}\right\}$ and each $s\in V\backslash \tilde{A_{i}}$, let $\delta_{i,s}=w_{i}\left(f_{i}\left(\tilde{A_{i}}\cup \left\{s\right\}\right)-f_{i}\left(\tilde{A_{i}}\right)\right)$. Let $r_{i,s}=\delta_{i,s}/c_{i,s}$ where $c_{i,s}$ denotes the incremental cost of adding the ith type of sensor to location s. Let $s_{1},s_{2},\cdots ,s_{m}$ be the sequence of locations with $r_{i,s}$ in descending order and $p_{1},p_{2},\cdots ,p_{m}$ be the sequence of selected types. Let k be such that $C=\sum_{i=1}^{k-1}c_{p_{i},s_{i}}\leq B$ and $\sum_{i=1}^{k}c_{p_{i},s_{i}}>B$. Let $\lambda =\left(B-C\right)/c_{p_{k},s_{k}}$. Then*

$$\max_{A,c(A)\leq B}\sum_{i=1}^{T}w_{i}f_{i}\left(A_{i}\right)\leq \sum_{i=1}^{T}w_{i}f_{i}\left(\hat{A_{i}}\right)+\sum_{i=1}^{k-1}\delta_{s_{i}}+\lambda \delta_{s_{k}}$$

### 3.3. Assessing the Trade Off

After we obtain the placement scheme A for the general case, we know the station number *K* and the number of sensors $k_{i}$ for the *i*th spatial field. If we run the simple greedy algorithm for the *i*th type of spatial field with subset size constraint $k_{i}$, we can find the uncoupled placement result $A_{i}^{\prime }$ with $|A_{i}^{\prime }|=k_{i}$ and obtain the individual sacrifice $f_{i}\left(A_{i}^{\prime }\right)-f_{i}\left(A_{i}\right)$ due to the budget constraint. The total information loss is $\sum_{i=1}^{T}\left(f_{i}\left(A_{i}^{\prime }\right)-f_{i}\left(A_{i}\right)\right)$ with a total saving of $c_{site}\cdot (\cup_{i=1}^{c}|A_{i}^{\prime }|-\cup_{i=1}^{c}\left|A_{i}^{\prime }\right|$). This information is useful to assess whether additional budgets should be allocated for a certain field to provide further information.

### 3.4. Speeding up the Algorithms

Krause et al. [8] developed a lazy evaluation technique to speed up the greedy selection algorithm for single-type sensor placement problem. In this section, we adapt this approach for the multi-type sensor placement problem to speed up the two algorithms proposed above.

We start with the one-with-all case. The key idea is that, at each iteration, some calculations of the information gain can be saved by utilizing the submodular property, i.e., the information gain for adding sensors can never increase. Therefore, we can maintain an ordered list of the information gain and only update the value when necessary. The lazy greedy for the this case is presented with Algorithm 3.

**Algorithm 3** Lazy greedy algorithm for one-with-all case
**Input:** Station number constraint *K*, a set of grids *V*, objective function $f_{1},f_{2},\cdots ,f_{T}$, weights $w_{1},w_{2},\cdots ,w_{T}$**Output:** A subset of locations A⊆V A=∅. for location *s*, calculate the initial incremental gain $\delta_{s}=\sum_{i=1}^{T}w_{i}f_{i}\left(\left\{s\right\}\right)$ $s^{*}=\arg\max\delta_{s}$, add $s^{*}$ to location set *A*. remove $\delta_{s^{*}}$ from $\delta_{s}$ and sort the gain $\delta_{s}$ in descending order $\left[\delta_{s^{1}},\delta_{s^{2}},\cdots \delta_{s^{n-1}}\right]$. **for** j=2 to *K* **do**  **while** $\sum_{i=1}^{T}w_{i}\left(f_{i}\left(A\cup \left\{s^{1}\right\}\right)-f_{i}\left(A\right)\right)<\delta_{s^{2}}$ **do**   update $\delta_{s^{1}}=\sum_{i=1}^{T}w_{i}\left(f_{i}\left(A\cup \left\{s^{1}\right\}\right)-f_{i}\left(A\right)\right)$.   sort $\delta_{s}$ in descending order $\left[\delta_{s^{1}},\delta_{s^{2}},\cdots \delta_{s^{n-1}}\right]$.  **end while**  add $s^{1}$ to location set *A* and remove $\delta_{s^{1}}$ from $\delta_{s}$. **end for** **return** *A*


The idea is similar for the general case. Specifically, for the cost-effective greedy selection, we maintain an ordered list of information gain per cost for adding a certain type of sensor to a specific location. Notice that the cost is also dependent on the existing selection and hence the cost update after each iteration (if any) will cause the reordering of the list. The lazy greedy for the general case is presented with Algorithm 4.

**Algorithm 4** Lazy greedy algorithm for the general cost case
**Input:** Budget *B*, a set of grid index *V*, objective function $f_{1},f_{2},\cdots ,f_{T}$, weights $w_{1},w_{2},\cdots ,w_{T}$, site construction cost $c_{site}$, sensor cost $c_{1},c_{2},\cdots ,c_{T}$**Output:** A placement scheme A⊂V $A^{\prime }=A^{\prime \prime }=\emptyset ,V^{\prime }=V^{\prime \prime }=V,B^{\prime }=B^{\prime \prime }=B$ for location *s*, calculate the initial incremental gain of type *i* sensor $\delta_{i,s}=w_{i}f_{i}\left(s\right)$ $i^{*},s^{*}=\arg\max\delta_{i,s}$, add $s^{*}$ to location set $A_{i}^{{}^{\prime }}$. remove $\delta_{i^{*},s^{*}}$ from the gain list $\delta_{i,s}$ and sort it in descending order $\left[\delta_{i^{1},s^{1}},\delta_{i^{2},s^{2}},\cdots \delta_{i^{nT-1},s^{nT-1}}\right]$. **while** the search space $V^{\prime }$ is not empty **do**  **while** $w_{i^{1}}\left(f_{i}\left(s^{1}\cup A_{i^{1}}^{{}^{\prime }}\right)-f_{i}\left(A_{i^{1}}^{{}^{\prime }}\right)\right)<\delta_{i^{2},s^{2}}$ **do**
   update $\delta_{i^{1},s^{1}}=w_{i^{1}}\left(f_{i}\left(s^{1}\cup A_{i^{1}}^{{}^{\prime }}\right)-f_{i}\left(A_{i^{1}}^{{}^{\prime }}\right)\right)$ and sort $\delta_{i,s}$ in descending order.  **end while**  **if** the incremental cost ${△}_{i^{1},s^{1}}c\left(A^{\prime }\right)$ is less than the remaining budget $B^{\prime }$ **then**   Update the remaining budget $B^{\prime }=B^{\prime }-{△}_{i^{1},s^{1}}c\left(A^{\prime }\right)$   Add location $s^{1}$ to the location set $A_{i^{1}}^{{}^{\prime }}$  **end if**  Remove location $s^{1}$ from search space $V_{i^{1}}^{{}^{\prime }}$ and $\delta_{i^{1},s^{1}}$ from the list **end while** for location *s*, calculate the initial incremental gain per cost of type *i* sensor $\delta_{i,s}=\frac{w_{i}f_{i}\left(s\right)}{{△}_{i,s}c\left(A^{\prime \prime }\right)}$ $i^{*},s^{*}=\arg\max\delta_{i,s}$, add $s^{*}$ to location set $A_{i}^{{}^{\prime \prime }}$. remove $\delta_{i^{*},s^{*}}$ from the list of $\delta_{i,s}$ and sort it in descending order $\left[\delta_{i^{1},s^{1}},\delta_{i^{2},s^{2}},\cdots \delta_{i^{nT-1},s^{nT-1}}\right]$. **while** the search space $V^{\prime \prime }$ is not empty **do**  **while** $\frac{w_{i^{1}}\left(f_{i}\left(s^{1}\cup A_{i^{1}}^{{}^{\prime \prime }}\right)-f_{i}\left(A_{i^{1}}^{{}^{\prime \prime }}\right)\right)}{{△}_{i,s}c\left(A^{\prime \prime }\right)}<\delta_{i^{2},s^{2}}$ **do**   update $\delta_{i^{1},s^{1}}=\frac{w_{i^{1}}\left(f_{i}\left(s^{1}\cup A_{i^{1}}^{{}^{\prime \prime }}\right)-f_{i}\left(A_{i^{1}}^{{}^{\prime \prime }}\right)\right)}{{△}_{i,s}c\left(A^{\prime \prime }\right)}$ and sort $\delta_{i,s}$ in descending order.  **end while**  **if** the cost ${△}_{i^{1},s^{1}}c\left(A^{\prime \prime }\right)$ is less than the remaining budget $B^{\prime \prime }$ **then**   Update the remaining budget $B^{\prime \prime }=B^{\prime \prime }-{△}_{i,s}c\left(A^{\prime \prime }\right)$   Add location $s^{1}$ to the location set $A_{i}^{\prime \prime }$   Update $\delta_{i,s}$ of the other type of sensors at location $s^{1}$ and sort the list  **end if**  Remove location $s^{1}$ from search space $V_{i}^{\prime \prime }$ and $\delta_{i^{1},s^{1}}$ from the list **end while** Select the better scheme A in terms of the objective value $A=\arg\max_{A\in \{A^{\prime },A^{\prime \prime }\}}\sum_{i=1}^{T}w_{i}f_{i}\left(A_{i}\right)$ **return** A


## 4. Simulations

**Figure 4:**
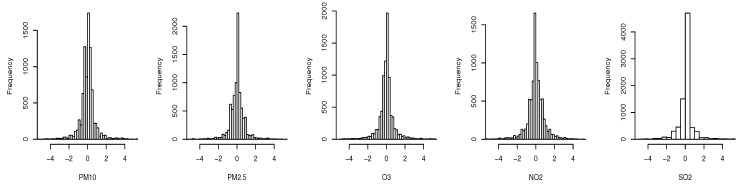
Histogram of the normalized one-hour difference of the hourly measurements at Tung Chung station over the year 2017.

We evaluated the proposed multi-type placement scheme on the hourly air quality monitoring data for 2017 provided by the Hong Kong Environment Protection Department (EPD) website [16]. There are 16 monitoring stations in total, three of which are roadside stations. Here, we only considered ${\mathrm{PM}}_{2.5}$, ${\mathrm{PM}}_{10}$, ${\mathrm{NO}}_{2}$, $O_{3}$ and ${\mathrm{SO}}_{2}$ as they are measured by all of the stations. As shown in Figure 4, the distribution of normalized one-hour difference of the pollutants at Tung Chung monitoring station are approximately normal distributed (due to space limits, we only show one station as an example; in fact, the statement holds for arbitrary station (location)), and hence satisfy the GP assumption. We estimated the empirical covariance matrix from the data, which represents the spatial process accurately. Here, we chose the entropy criterion as it directly satisfies the monotone and submodular property without the further requirement of that the number of sensors available is much smaller than the total number of possible locations. Let $X_{s}^{\left(i\right)}$ denote the distribution of the *i*th field at location s. Then, the incremental information gain of adding a sensor of type *i* to location *s* is

$$\begin{array}{c} \delta_{i,s}=H\left(X_{s}^{\left(i\right)}|X_{A}^{\left(i\right)}\right)=\frac{1}{2}\log\left(2\pi e\sigma_{X_{s}^{\left(i\right)}|X_{A}^{\left(i\right)}}^{2}\right) \end{array}$$

**Figure 5:**
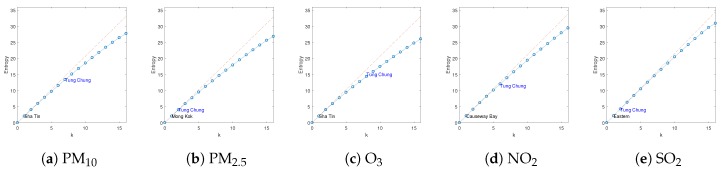
Comparison of information gain of different spatial fields with simple greedy selection.

Figure 5 compares the information gain of different spatial fields using simple greedy selection. The blue line represents the objective function and the orange line is a straight line with a slope of maximum individual entropy. As can be seen, all objective functions exhibit the diminishing returns property and it is natural to expect a more prominent effect when the number of sensors is much larger. The name of the first location selected is displayed in each of the objective. Specifically, we know that Causeway Bay is the most uncertain location in terms of ${\mathrm{NO}}_{2}$, which is likely due to the car emissions, and Sha Tin is the most uncertain location in terms of ${\mathrm{PM}}_{10}$ and $O_{3}$. We also chose Tung Chung as a representative and display when it is selected in each field, which clearly indicates that each spatial field has different properties.

**Figure 6:**
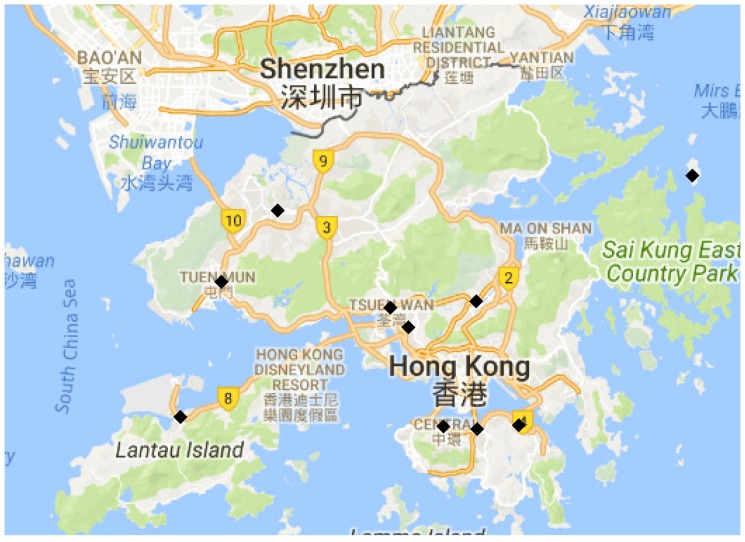
Placement results of 10 sensors in Hong Kong for one-with-all case.

Figure 6 shows the placement results for the one-with-all case when k=10. The weights $w_{i},i=1,2,\cdots ,5$ were set to be identical for each field. Different from the single best location for each field, Central was selected first here as it is the most uncertain location with respect to the sum of individual objectives.

**Figure 7:**
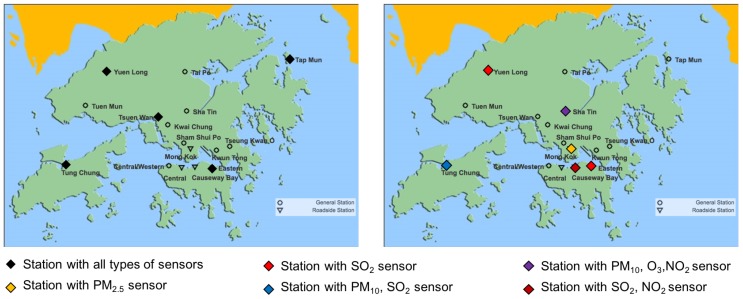
Placement results for the general case when the budget is 100, the cost is $c_{1}=c_{2}=c_{3}=c_{4}=c_{5}=1$, $c_{site}=15$. The left figure is the placement result with the cost-effective greedy selection. The right figure is the placement result with the greedy selection.

Figure 7 shows the placement results for the general case when the budget is just enough to deploy five stations, each with all types of sensors. The weights $w_{i},i=1,2,\cdots ,5$ were set to be identical for each field. As shown in the figure, the cost-effective greedy approach is able to make full use of the budget and selects five locations for deploying stations with all type of sensors. The greedy approach, however, picks six locations for deploying stations yet none of them has all type of sensors due to the lack of consideration of cost during the selection. Since much more sensors are deployed with the cost-effective greedy approach as compared to the greedy approach (25 vs. 9), the total information gain of the cost-effective greedy approach is larger. Therefore, the the final placement result of the hybrid greedy approach is proposed by the cost effective greedy selection in this case.

**Figure 8:**
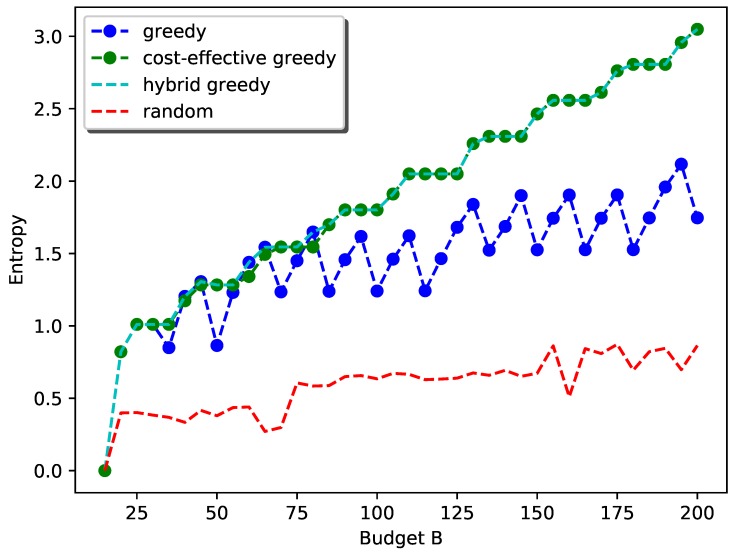
Performance of the hybrid greedy approach with equal weight $w_{i}$.

Figure 8 shows the performance of the proposed hybrid greedy approach for the general case. We used the random selection as the baseline for comparison. The performance of simple-greedy selection and cost-effective greedy selection are also shown. Here, we set $c_{1}=2$, $c_{2}=2$, $c_{3}=1$, $c_{4}=1$, $c_{5}=1$, $c_{site}=15$ as the cost of PM sensors are generally higher and vary *B* from 15 to 200 at a step size of 5. The weights $w_{i},i=1,2,\cdots ,5$ are set to be identical for each field.

As can be seen from the plot, as the budget goes up, the general trend of information gain with the hybrid greedy approach follows the submodular property. The flat regions in the curve correspond to the scenarios when the general cost constraint can be reduced to the simple cardinality constraint. For example, when the budget is 25 to 30, $k_{\max}=k_{\min}=1$ and the strategy is simply deploying one station with all types of sensors.

For the cost-effective greedy selection, the increase is steady as the placement strategy in this scenario will first select a location for one type and then deploy other types at that location until the budget allows for a new station. The reason is that the site cost is quite high compared to the sensor cost and it is not cost effective to deploy a new station when sensors can still be added to the existing stations. This also explains the flat region even when $k_{\min}<k_{\max}$.

The simple greedy selection, however, has a sudden drop when the budget first allows for a new station. The reason is that this approach will prefer exploring a new location with larger information gain rather than using the budget for adding other sensors to existing stations with smaller information gain and hence less sensors can be added.

**Figure 9:**
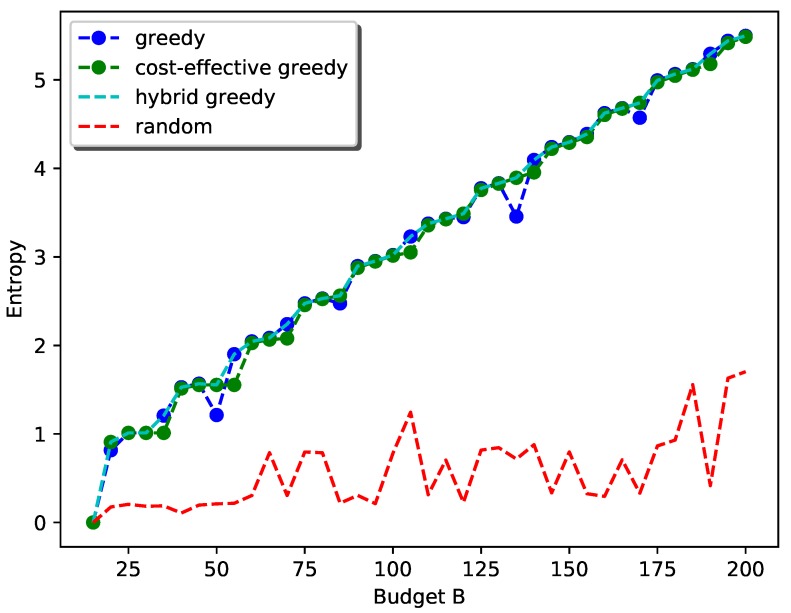
Performance of the hybrid greedy approach with a higher weight for ${\mathrm{PM}}_{2.5}$.

Figure 9 shows the performance of the proposed hybrid greedy approach for the general case with a special emphasis on ${\mathrm{PM}}_{2.5}$ as it is the most health-harmful air pollutant [27]. Here, we set the weight for ${\mathrm{PM}}_{2.5}$ to 0.6 and other weights to 0.1. The other settings remain the same. As can be seen from the plot, the performance of simple-greedy and cost-effective greedy are comparable due to the high weight of the objective function for ${\mathrm{PM}}_{2.5}$. This is because adding ${\mathrm{PM}}_{2.5}$ sensors greedily without considering the cost will still increase the total information gain. Furthermore, the flat regions for cost-effective greedy disappear, indicating it will be preferable to deploy new stations for ${\mathrm{PM}}_{2.5}$ than to add sensors to existing stations.

Both plots show that the proposed hybrid greedy approach always performs much better than random selection in terms of total information gain.

**Figure 10:**
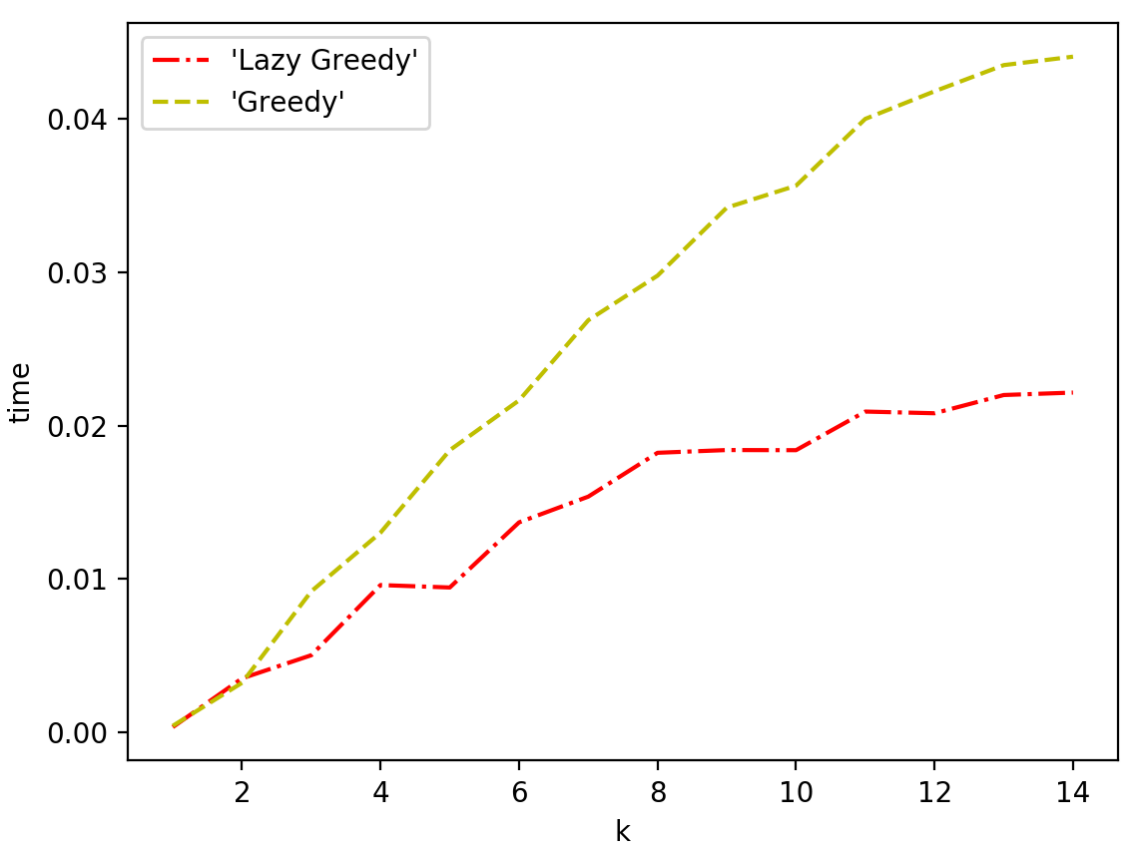
Greedy vs. lazy greedy for one-with-all case.

**Figure 11:**
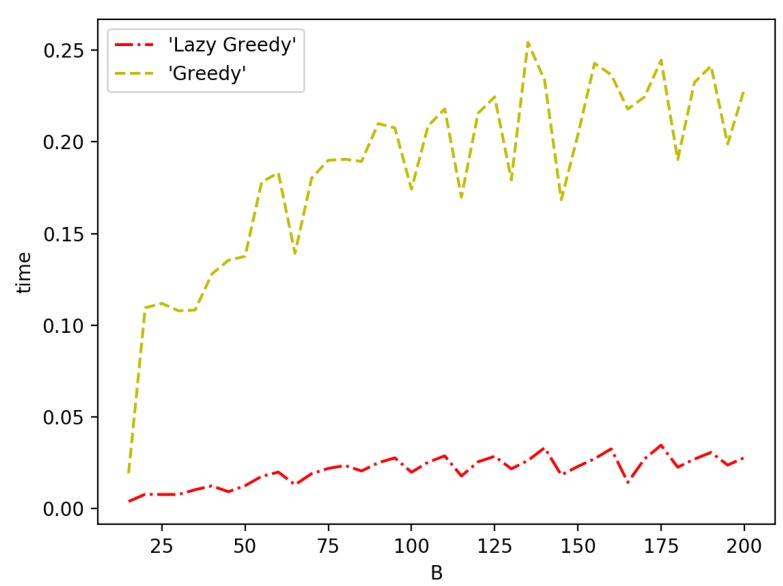
Greedy vs. lazy greedy for general case.

Figure 10 and Figure 11 show the speed performance comparison of greedy approach with lazy greedy approach. It can be easily seen that lazy greedy is faster than greedy while achieving the same approximation guarantee. For the general case, there is a more significant improvement. The reason is that, at each iteration, the candidate pool of possible selections is larger and hence more function evaluations can be saved with a lazy approach.

## 5. Conclusions

In this paper, we formulate the multi-type sensor placement problem in Gaussian spatial field for environmental monitoring. We analyze two cases with different assumptions on the station requirement and propose two greedy algorithms with approximation guarantees. We then introduce a lazy approach for speeding up the greedy algorithms while achieving the same performance guarantee. We evaluated the proposed approach via an application in air quality monitoring scenario in Hong Kong and experimental results demonstrate the effectiveness of the proposed approach. This formulate can provide guidance for designing a citywide multi-type sensor network for environmental monitoring cost-effectively. 

## Figures and Tables

**Table 1:** Notations.

Notation	Definition
m(·)	The mean function of the Gaussian Process
k(·,·)	The kernel function of the Gaussian Process
$X_{A}$	The random variables over the location index set *A*
σ	Try to span the whole column of the table
*T*	The total number of types of interest
[T]	The abbreviation for the set {1,2,⋯,T}
*V*	The set of all indexes, each corresponding to a location/grid
|V|	The number of indexes in the set *V*
*s*	an index in the set *V*
$A_{i}$	The set of the indexes of the selected locations for the *i*th type
A	The placement scheme $\left\{A_{1},A_{2},\cdots ,A_{T}\right\}$
$f_{i}$	The *i*the objective function
$w_{i}$	The weight parameter of the *i*th objective function
$c_{i}$	The unit cost for the *i*th type
$c_{site}$	The site construction cost
*B*	The total budget constraint
*K*	The subset size constraint
$k_{i}$	The total number of sensors for the *i*th type
⌊x⌋	The floor function mapping *x* to the greatest integer
	less than or equal to *x*
$\delta_{i,s}$	The information gain of adding location index *s* of type *i*
